# Dual rare genetic diseases in five pediatric patients: insights from next-generation diagnostic methods

**DOI:** 10.1186/s13023-024-03148-3

**Published:** 2024-04-12

**Authors:** Yupeng Liu, Xue Ma, Zhehui Chen, Ruxuan He, Yao Zhang, Hui Dong, Yanyan Ma, Tongfei Wu, Qiao Wang, Yuan Ding, Xiyuan Li, Dongxiao Li, Jinqing Song, Mengqiu Li, Ying Jin, Jiong Qin, Yanling Yang

**Affiliations:** 1 Department of Pediatrics, Peking University People’s Hospital, Beijing, China; 2 Department of Pediatrics, Peking University First Hospital, Beijing, China; 3 Department of Respiration, Beijing Children’s Hospital, Capital Medical University, Beijing, China; 4 Department of Pediatrics, Qinghai University Affiliated Hospital, Xining, China; 5 Department of Endocrinology, Genetics and Metabolism, Beijing Children’s Hospital, Capital Medical University, Beijing, China; 6 Department of Precise Medicine, General Hospital of Tianjin Medical University, Tianjin, China; 7 Children’s Hospital Affiliated to Zhengzhou University, Zhengzhou, China

**Keywords:** Genetic disease, Dual molecular diagnoses, Whole-exome sequencing, Methylmalonic acid

## Abstract

**Background:**

Clinicians traditionally aim to identify a singular explanation for the clinical presentation of a patient; however, in some cases, the diagnosis may remain elusive or fail to comprehensively explain the clinical findings. In recent years, advancements in next-generation sequencing, including whole-exome sequencing, have led to the incidental identification of dual diagnoses in patients. Herein we present the cases of five pediatric patients diagnosed with dual rare genetic diseases. Their natural history and diagnostic process were explored, and lessons learned from utilizing next-generation diagnostic technologies have been reported.

**Results:**

Five pediatric cases (3 boys, 2 girls) with dual diagnoses were reported. The age at diagnosis was from 3 months to 10 years. The main clinical presentations were psychomotor retardation and increased muscular tension, some accompanied with liver dysfunction, abnormal appearance, precocious puberty, dorsiflexion restriction and varus of both feet, etc. After whole-exome sequencing, nine diseases were confirmed in these patients: Angelman syndrome and Krabbe disease in case 1, Citrin deficiency and Kabuki syndrome in case 2, Homocysteinemia type 2 and Copy number variant in case 3, Isolated methylmalonic acidemia and Niemann-Pick disease type B in case 4, Isolated methylmalonic acidemia and 21-hydroxylase deficiency in case 5. Fifteen gene mutations and 2 CNVs were identified. Four novel mutations were observed, including c.15292de1A in *KMT2D*, c.159_164inv and c.1427G > A in *SLC25A13*, and c.591 C > G in *MTHFR*.

**Conclusions:**

Our findings underscore the importance of clinicians being vigilant about the significance of historical and physical examination. Comprehensive clinical experience is crucial for identifying atypical clinical features, particularly in cases involving dual rare genetic diseases.

## Background

In the realm of rare genetic diseases, ensuring effective medical management and genetic counseling hinges on providing precise diagnoses. However, clinical evaluations and conventional genetic testing bring about the diagnosis in < 50% patients [1]. The capacity to offer molecular diagnostics to patients has witnessed a substantial improvement, rapidly emerging as the most suitable method for those with rare diseases. Consequently, it has become an indispensable process for identifying various genetic diseases in a single patient [2].

A distinctive facet of the dual molecular diagnostic process is the occurrence of a unique or overlapping clinical diagnosis involving > 1 independently genetically separated locus. Medical literature intermittently features reports on patients with concomitant diagnoses of ≥ 2 genetically related disorders, colloquially known as “double trouble” [3]. The identification of a significant number of cases with dual genetic diseases is incidental, often occurring during next-generation or whole sequencing procedures [2–4]. Whole-exome sequencing data indicate that the prevalence of dual molecular diagnosis, contrary to previous assumptions, may be higher, readily detectable in approximately 7% cases [2]. Herein we present a description of the clinical evaluation of five patients with dual molecular diagnoses of genetic diseases.

## Methods

From 2013 to 2022, 1,659 patients were diagnosed at our hospitals with inherited metabolic disorders via gene sequencing. In this retrospective study, we aimed to evaluate five pediatric patients exhibiting dual rare genetic diseases observed in our outpatient clinics. The comprehensive data collection encompassed age at onset and diagnosis, clinical manifestations, family history, treatment, and outcomes. In addition, routine blood and urine examination was performed to assess liver, renal, and heart functions.

Dried blood spots were collected for further analyses. As previously described, liquid chromatography–mass spectrometry (Waters MS/MS system A, 1445–002; API3200, Applied Biosystems, CA, USA) was employed to analyze blood amino acids, free carnitine, and acylcarnitines [5–7]. Metabolite concentrations were computed using ChemoView software. Urinary organic acids were analyzed using gas chromatography–mass spectrometry, which was performed on a Shimadzu GCMS-QP2010 system (Kyoto, Japan) [8–10]. Fluorescence polarization immunoassay was utilized to analyze plasma total homocysteine levels. Lysosomal enzyme activity in peripheral blood leukocytes was determined using a synthetic fluorescent substrate based on a standard curve.

Genomic DNA from peripheral blood samples was extracted, purified, and subsequently sent to Euler Genomics (Beijing, China), Berry Genomics Corporation (Beijing, China), and GrandOmics (Beijing, China) for next-generation or whole-exon sequencing for variant screening. DNA samples were sequenced on Illumina HiSeq 2500 (Illumina, San Diego, USA). Each variant was compared with information from 1000 Genomics (www.1000genomes.org), the ExAC database (http://exac.broadinstitute.org/), and the gnomAD database (http://gnomad.broadinstitute.org/). *In silico* analysis of variants to predict pathogenicity also involved Mutation Taster, PolyPhen-2, and SIFT. Interpretation of the variants was based on the Human Gene Mutation Database (http://www.hgmd.cf.ac.uk/ac/index.php). Pathogenicity assessment followed the American College of Medical Genetics and Genomics guidelines, classifying variants as pathogenic, likely pathogenic, variants of uncertain significance, likely benign, or benign.

Before research commencement, a consent form was signed by all patients or their caregivers, indicating their approval for all aspects of the clinical assessment, including genetic testing, and all ancillary testing (e.g., bone marrow aspiration).

### Clinical reports

#### Case 1: Angelman syndrome and krabbe disease

Case 1, a 7-year-old boy, presented with general developmental delay, irritability, and a distinctive giggle. Physical assessment revealed muscle tension, limb stiffness, thumb buckling, and substandard comprehension proficiency. Brain MRI revealed myelin dysplasia, while blood amino acid and acylcarnitine profiles were normal. Electroencephalogram data indicated slow wave emission from bilateral occipital and temporal spines. Chromosome G banding was 46, XY. Whole-exome sequencing identified a 4.88 Mb deletion in 15q11.2q13.1 (copy number variation, CNV) and compound heterozygous variants, c.1963G > A (p.V655M) and c.1511T > C (p.L504P), in the GALC gene. The CNV was associated with Angelman syndrome, while the two GALC variants were found to originate from his parents (Tables 1, 2 and 3). Peripheral leukocyte galactose cerebrosidase activity showed a significant decrease (3.8 nmol/g/h, normal range 33.4–123.9 nmol/g/h), confirming the diagnosis of Krabbe disease.

**Table 1:** Clinical features of five pediatric patients with dual genetic diseases

Clinical features	Patient 1	Patient 2	Patient 3	Patient 4	Patient 5
Gender	M	F	M	F	M
Age at diagnosis	7 years	3 months	10 years	3 years	1 year and 8 months
Genetic diseases	Angelman syndrome	Citrin deficiency	Homocysteinemia type 2	Isolated methylmalonic acidemia	Isolated methylmalonic acidemia
	Krabbe disease	Kabuki syndrome	Copy number variant	Niemann-Pick disease type B	21-hydroxylase deficiency
Neurology	General developmental delay, increased muscle tension, poor grasping	Developmental delay, increased muscular tension	Severe mental delay, seizures, dysarthria, increased muscle tension	General developmental delay	Psychomotor retardation
Skeletal and muscle	Thumb buckling	-	Dorsiflexion restriction and varus of both feet	-	-
Digestive	-	Liver dysfunction	-	Abnormal liver function	Feeding difficulties, diarrhea, metabolic acidosis
Appearance	-	Long cleft on the eyelid, elongated lateral canthus, heavy eyebrows, cleft palate	-	-	-
Endocrine	-	-	-	-	Precocious puberty
Other features	Irritability and a distinctive giggle	-	-	-	-
Genes with pathogenic variants					
Gene A	15q11.2q13.1del	KMT2D	16p11.2del	MMUT	MMUT
Gene B	GALC	SLC25A13	MTHFR	SMPD1	CYP21A2
Modes of inheritance (Gene A + Gene B)	AD + AR	AD + AR	AD + AR	AR + AR	AR + AR

**Table 2:** Novel variants associated with patient conditions

Gene	Nucleotide substitution	Amino acid substitution	Mutation taster	SIFT	Polyphen-2	Mutation assessor	GERP++	1000G	ExAC	gnomAD
SLC25A3	c.1427G > A	p.R476Q	Disease_causing	Damaging	Benign	Medium	3.75	-	0.0000160	0.0000122
MTHFR	c.591C > G	p.Y197*	Disease_causing	-	-	-	4.07	-	-	-

Note: Pathogenicity analysis was done as per ACMG standards

**Table 3:** Genotype profiles of five patients

Patient	Gene	Nucleotide substitution	Amino acid substitution	Parental origin	Novel/Reported	Pathogenicity
Case 1	15q11.2q13.1del	-	-	De novo	Reported, PMID 34203304	Pathogenic
	GALC	c.1963G > A	p.V655M	Father	Reported, PMID 31885218	Pathogenic
		c.1511T > C	p.L504P	Mother	Reported, PMID 28598007	Pathogenic
Case 2	KMT2D	c.15292de1A	p.T5098Lfs*49	De novo	Novel	Pathogenic
	SLC25A13	c.159_164inv	p.N54delinsD*	Father	Novel	Pathogenic
		c.1427G > A	p.R476Q	Mother	Reported, ClinVar 2073364	Pathogenic
Case 3	16p11.2del	-	-	De novo	Reported, PMID 35595456	Pathogenic
	MTHFR	c.591C > G	p.Y197*	Father	Novel	Pathogenic
		c.584G > A	p.A195V	Mother	Reported, PMID 21132537	Pathogenic
Case 4	MMUT	c.323G > A	p.R108H	Father	Reported, PMID 11528502	Pathogenic
		c.1540C > A	p.Q514K	Mother	Reported, PMID 26454439	Pathogenic
	SMPD1	c.1144C > T	p.L382F	Father	Reported, PMID 23356216	Pathogenic
		c.1675G > T	p.V559L	Mother	Reported, PMID 23356216	Pathogenic
Case 5	MMUT	c.866G > C	p.Y289T	Father	Reported, PMID 31622506	Pathogenic
		c.2179C > T	p.R727X	Mother	Reported, PMID 34668645	Pathogenic
	CYP21A2	c.188A > T	p.H63L	Father	Reported, PMID 18319307	Pathogenic
		c.518T > A	p.I173N	Mother	Reported, PMID 3257825	Pathogenic

Note: Pathogenicity analysis was done as per ACMG standards

#### Case 2: Citrin deficiency and kabuki syndrome

Case 2, a girl, visited us with developmental delay and liver dysfunction. Born at term (weight, 3.3 kg) after an uncomplicated pregnancy, she exhibited persistent jaundice until 3 months of age. Elevated levels of serum alpha-fetoprotein and blood citrulline (110 µmol/L, normal range 10–50 µmol/L) were observed, along with mild liver dysfunction and hyperammonemia. Ultrasound of the abdomen revealed hepatomegaly, but brain MRI was normal. Treatment with a lactose-free diet led to gradual improvement, with liver function and blood citrulline level returning to normal after 6 months.

Physical examination revealed distinctive features, such as a long cleft on the eyelid, elongated lateral canthus, heavy eyebrows, cleft palate, and increased muscle tension. Chromosome G banding was 46, XX. Whole-exome sequencing identified a *de novo* variant c.15292de1A (p.T5098Lfs*49) in the KMT2D gene, supporting the diagnosis of Kabuki syndrome. Besides, compound heterozygous variants in the SLC25A13 gene, c.159_164inv (p.N54delinsD*) and c.1427G > A (p.R476Q), were identified. Her parents were heterozygous carriers at the same mutation site in SLC25A13. These findings confirmed the second diagnosis of citrin deficiency.

#### Case 3: homocysteinemia type 2 and copy number variant

A 10-year-old boy visited our hospital with psychomotor retardation and seizures. He exhibited normalcy during the newborn period. However, developmental delays in major motor skills surfaced after one month of age, with independent walking achieved at 3 years. A progressive deterioration in motor functions emerged at the age of 8 years, characterized by dysarthria, increased muscular tension in both lower limbs, dorsiflexion restriction, and varus of both feet, which were identified upon physical assessment. MRI indicated brain atrophy. Urinary organic acid, blood amino acid, and acylcarnitine profiles were normal, but plasma total homocysteine level was increased to 108.5 µmol/L (normal range 0–15 µmol/L) and 5-methyltetrahydrofolate level in cerebrospinal fluid was significantly decreased to 19.8 nmol/L (normal range 60–210 nmol/L), indicative of secondary cerebral folate deficiency. Compound heterozygous variants, c.591C > G (p.Y197*) and c.584G > A (p.A195V), in the MTHFR gene confirmed the diagnosis of homocysteinemia type 2 due to methylenetetrahydrofolate reductase deficiency. Simultaneously, whole-exome sequencing identified a 0.648 Mb deletion in 16p11.2, confirming the diagnosis of 16p11.2 deletion syndrome. Both parents were found to carry heterozygous MTHFR variants without the deletion in 16p11.2.

#### Case 4: Isolated methylmalonic acidemia and niemann-pick disease type B

A 3-year-old girl visited our hospital with general developmental delay. Initially considered for cerebral palsy at 1 year of age, she underwent physical rehabilitation. Routine blood test results were normal, but significantly increased levels of urine methylmalonate and methylcitrate were observed. Blood propionyl carnitine level was increased to 15.2 µmol/L (normal range 1.0–5.0 µmol/L), and propionylcarnitine/acetylcarnitine ratio was high at 0.53 (normal range 0.03–0.25). Plasma total homocysteine level was normal. These findings led to the diagnosis of isolated methylmalonic aciduria (MMA). Whole-exome sequencing identified compound heterozygous variants in methylmalonyl-CoA mutase (MMUT, c.1540C > A and c.323G > A), confirming the diagnosis of MMUT deficiency.

Clinical examination also revealed liver abnormalities. Serum alanine aminotransferase (59.4 IU/L) and aspartate aminotransferase (73.7 IU/L) levels showed a slight increase. Abdominal ultrasonography revealed significant enlargement of the liver (lower edge 3.1 cm below the right costal margin) and spleen (lower edge 2.2 cm below the left costal margin). Whole-exome sequencing identified compound heterozygous variants (c.1144C > T and c.1675G > T) in SMPD1 gene. Niemann-Pick cells were found in her bone marrow. Further, peripheral leukocyte acid sphingomyelinase activity was significantly decreased (80.8 nmol/g/min, normal value 216.1–950.9 nmol/g/min), confirming the diagnosis of Niemann-Pick disease type B.

#### Case 5: Isolated methylmalonic acidemia and 21-hydroxylase deficiency

A boy, aged 1 year and 8 months, visited us with feeding difficulties, diarrhea, metabolic acidosis, and psychomotor retardation following vaccination against polio at 3 months of age. He exhibited a significant increase in urine methylmalonate (34.4 mmol/mol creatinine, normal range 0.2–3.6 mmol/mol creatinine) and methylcitrate levels, while plasma total homocysteine level was normal. Two pathogenic variants (c.866G > C and c.2179C > T) in MMUT gene supported the diagnosis of methylmalonyl-CoA mutase deficiency. Treatment with cobalamin intramuscular injection and L-carnitine supplementation improved his clinical picture. Mental development and upper limb motor function normalized, but spastic paralysis persisted in the lower limbs, necessitating the use of a brace.

At 5 years of age, signs of precocious puberty emerged. His left wrist bone age was 13 years. Serum growth hormone level was normal. Upon gonadotropin-releasing hormone stimulation, the luteinizing hormone/follicle stimulating hormone peak value was < 0.6. Plasma adrenocorticotropic hormone levels showed a marked increase (309 pg/mL, normal range 0–46 pg/mL), while plasma cortisol levels were normal (7.9 µg/dL, normal range 5–25 µg/dL). Testosterone and 17-α-hydroxyprogesterone levels were increased to 5.77 ng/mL (normal range 0.27–0.90 ng/mL) and 495 nmol/L (normal control < 30 nmol/L), respectively. Estradiol level was normal at < 20 pg/mL (normal range 0–56 pg/mL). Aldosterone level was 32.29 ng/dL (normal range 5–17.5 ng/dL in horizontal position and 6.5–30 ng/dL in vertical position). MRI revealed no abnormalities in the pituitary and hypothalamus. Notably, double adrenal ultrasound, along with CT enhancement, depicted bilateral adrenal thickening, particularly on the left. Endocrine examination results indicated congenital adrenal hyperplasia due to 21-hydroxylase deficiency. Two pathogenic variants (c.188A > T and c.518T > A) were found in CYP21A2 gene, confirming the diagnosis.

## Discussion

In clinical training, clinicians are traditionally oriented toward identifying a single explanation for the clinical presentation of a patient. The concept of multiple genetic diseases in a single patient is perceived as complex and challenging [1]. Conventionally, clinicians abstract a specific phenotype to recognize Mendelian disease patterns, forming the basis for adequate diagnosis. Clinical attributes not aligning with this genetic etiology pattern may be considered phenotypic expressions. However, literature reports several cases of co-occurring genetic disorders [2–4]. Posey et al. indicated that the occurrence of molecular “double trouble” is not as rare as previously assumed, suggesting up to 7% patients diagnosed by whole-exome sequencing possess a dual or triple genetic diagnosis [2]. This challenges the hypothesis that a single diagnosis concludes genetic investigations. Complete diagnosis in such cases is challenging, as distinguishing whether atypical clinical features represent a novel primary disease phenotype or a second genetic or acquired disease poses difficulty.

Herein all five patients exhibited clinical features that overlapped with their primary diagnoses. However, the presence of atypical clinical features prompted secondary diagnoses. For instance, in Case 1, a distinctive giggle and general developmental delay could be attributed to Angelman syndrome, but irritability and skeletal/muscle presentations were untypical. In addition to Angelman syndrome, whole-exome sequencing and CNV analyses identified a compound heterozygous variant in the GALC gene; moreover, a significant decrease was observed in galactose cerebrosidase activity. These findings confirmed the diagnosis of Krabbe disease. Similar methods were applied in Cases 2, 3, and 4 for addressing non-specific presentations. As indicated in Table 1, the examination of a secondary disease for atypical features of the primary disease was systematically initiated. In Case 5, however, because of a variation in clinical symptomatology and a less specific physical assessment, secondary disease examination was relatively complex. At 3 months of age, Case 5 presented with feeding difficulties, diarrhea, metabolic acidosis, and psychomotor retardation following vaccination against polio. Elevated levels of urine methylmalonate and methylcitrate were observed, while total homocysteine level was normal. The diagnosis of isolated MMA was confirmed by Sanger sequencing of the MMUT gene. Clinical condition of this patient improved post-treatment. The emergence of precocious puberty at age 5 raised questions about whether it represented a new phenotype of isolated MMA or resulted from a second genetic or acquired disease. To address this, whole-exome sequencing was employed for a second diagnosis, confirming 21-hydroxylase deficiency. The significance of conducting accurate diagnoses is paramount for patients, families, and caregivers as precise diagnosis offers insights into patient condition from the perspective of natural history and prognosis, enabling the application of tailored therapeutic strategies. In the context of genetic conditions, this also ensures the provision of genetic counseling. When faced with an indistinct phenotype or possibility of various etiologies, comprehensive investigations become imperative. Employing advanced genome-wide techniques, such as chromosomal microarray analysis, can facilitate CNV identification; moreover, whole-exome sequencing can be applied to detect mutations in protein-coding genes.

MMA, a rare inherited disorder, is the most common organic aciduria in Mainland China [11, 12]. MMAs encompass a group of genetically heterogeneous autosomal recessive disorders stemming from defective metabolic pathways involving MMUT or its cofactor, cobalamin [13]. The clinical manifestation of MMA is intricate, ranging from asymptomatic disease to severe multisystem injuries and even death. MMAs tend to occur at any age, from prenatal to adult life [14, 15]. From June 1998 to December 2020, our hospital diagnosed 1,266 cases of MMA in Mainland China [11, 16, 17]. Predominant clinical presentations included psychomotor retardation and metabolic crisis [11, 18]. Multiorgan damage, including hematological abnormalities, pulmonary hypertension, kidney damage, eye disease, and skin lesion, was evident. Notably, megaloblastic anemia was a primary hematological abnormality [11, 16]. Cases 3 and 5 exhibited additional complexities, including liver function abnormalities and precocious puberty. Unfortunately, dual diagnoses were identified in these cases through whole-exome sequencing, underscoring the necessity for detailed history and physical examination in clinical practice.

It is conceivable that clinical signs and symptoms of multiple appropriately characterized disorders may exhibit similar biochemical and radiological attributes. This can lead to the masking and/or overlooking of one disorder if another affecting a similar system is present. Even if an atypical feature or pattern is identified, it is often attributed to the primary diagnosis, with secondary diagnoses potentially being overlooked. Consequently, the atypical feature of one disease may inaccurately reflect another undiagnosed condition, emphasizing the need for careful consideration of the complete assessment.

In cases where the clinical picture is atypical or exhibits more severity than expected, the possibility of a dual diagnosis (“double trouble”) should be contemplated [3]. Recent cases highlight the ongoing necessity for meticulous clinical evaluation, including a thorough physical examination, particularly in patients with atypical clinical features. The identification of another condition can significantly influence genetic management and counseling [2, 3].

## Conclusions

In conclusion, this study reports five pediatric cases with dual diagnoses confirmed through whole-exome sequencing. Our findings underscore the importance of considering the possibility of genetic diseases when the overall clinical picture does not align with a single unifying diagnosis. Clinicians are urged to be cognizant of the significance of physical examination and history, drawing on their extensive experience in identifying atypical clinical features.

## Data Availability

All data generated or analyzed during this study are included in this article.
